# Low-Velocity Impact Resistance and Compression After Impact Strength of Thermoplastic Nanofiber Toughened Carbon/Epoxy Composites with Different Layups

**DOI:** 10.3390/polym16213060

**Published:** 2024-10-30

**Authors:** Timo Meireman, Erik Verboven, Mathias Kersemans, Wim Van Paepegem, Karen De Clerck, Lode Daelemans

**Affiliations:** Department of Materials, Textiles and Chemical Engineering (MATCH), Ghent University, Technologiepark 907, B-9052 Zwijnaarde, Belgiummathias.kersemans@ugent.be (M.K.); wim.vanpaepegem@ugent.be (W.V.P.); karen.declerck@ugent.be (K.D.C.)

**Keywords:** damage resistance, electrospinning, residual compressive strength

## Abstract

This study investigates the effectiveness of polyether block amide (PEBA) thermoplastic elastomeric nanofibers in reducing low-velocity impact damage across three carbon fiber composite lay-up configurations: a cross-ply [0°/90°]2s (CP) and a quasi-isotropic [0°/45°/90°/−45°]s (QI) lay-up utilizing unidirectional plies, and a stacked woven [(0°,90°)]4s (W) lay-up using twill woven fabric plies. The flexural strength and interlaminar shear strength of the composites remained unaffected by the addition of nanofibers: around 750 MPa and 63 MPa for CP, 550 MPa and 58 MPa for QI, and 650 MPa and 50 MPa for W, respectively. The incorporation of nanofibers in the interlaminar regions resulted in a substantial reduction in projected damage area, ranging from 30% to 50% reduction over an impact energy range of 5–20 J. Microscopic analysis showed that especially the delamination damage decreased in toughened composites, while intralaminar damage remained similar for the cross-ply and quasi-isotropic lay-ups and decreased only in the woven lay-up. This agrees with the broad body of research that shows that interleaved nanofibers result in a higher delamination resistance due to toughening mechanisms related to nanofiber bridging of cracks. Despite their ability to mitigate delamination during impact, nanofibers showed limited positive effects on Compression After Impact (CAI) strength in quasi-isotropic and cross-ply composites. Interestingly, only the woven fabric composites demonstrated improved CAI strength, with a 12% improvement on average over the impact energy range, attributed to a reduction in both interlaminar and intralaminar damage. This study indicates the critical role of fiber integrity over delamination size in determining CAI performance, suggesting that the delaminations are not sufficiently large to induce buckling of sub-layers, thereby minimizing the effect of nanofiber toughening on the CAI strength.

## 1. Introduction

Fiber-reinforced composites are susceptible to significant loss of structural integrity when subjected to out-of-plane impact events, such as those caused by accidentally dropped tools, hail, or runway debris [1,2,3,4,5]. These impact events can result in severe fiber-matrix debonding and delamination of the reinforcing plies. Such damage is often barely visible externally, yet it can substantially reduce the residual compressive strength of the composite due to severe internal damage that is not discernible to the naked eye. The impact performance of composites is largely governed by the mechanical properties of the fibers, the matrix, and the interactions at their interfaces. Energy dissipation during impact primarily occurs through the formation of delaminations, matrix cracking, and fiber rupture, leading to significant reductions in residual strength [2,3,6,7], which is typically assessed through Compression After Impact (CAI) testing.

Improving impact resistance can thus significantly enhance the service life, safety, and maintenance costs of composites. The damage resistance of composite laminates under impact loading is often closely linked to the material’s delamination resistance [8,9,10,11,12,13,14,15,16,17,18,19]. In particular, high interlaminar fracture toughness is crucial, as delamination between plies is one of the main damage mechanisms that occur since the matrix-rich interlaminar regions are inherently weaker and experience large stress mismatches between adjacent plies. Hence, by improving the toughness of these regions and improving the delamination resistance, impact damage can be mitigated [19,20,21]. This is especially relevant for thermoset-based composites where the polymer matrix is typically less tough than in their thermoplastic counterparts.

Among different interlaminar toughening techniques, the use of electrospun nanofibrous interleaves has gained a lot of attraction in the past decade in the scientific literature. These interleaves are relatively simple to incorporate in composite laminates as they can be placed between reinforcing plies during manufacturing. The nanofibers themselves typically remain present within the interlaminar regions, where they form a toughened interlayer consisting of nanofibers embedded in the polymer matrix. There is a large body of research that has proven this to be a very effective way of improving the delamination resistance of laminates; see, for example, [22,23,24,25,26]. The primary toughening mechanism identified is the formation of nanofiber bridging zones upon impact, which effectively mitigates damage [21].

As the delamination resistance is considered to be an important factor that affects the performance of laminates under impact loadings, considering such loadings is a logical next step. Although the literature on the effects of nanofiber toughening on composite impact properties remains relatively limited [6,9,19,20,21,27,28,29,30,31,32,33,34,35], existing studies consistently indicate that polymer nanofibers can significantly reduce the damage area [1,6,19,20,21,28,29,30,31,32]. The main factor of this improved damage resistance is the improved interlaminar fracture toughness, which hinders the growth of delaminations under impact loadings.

Despite the ability of nanofibrous interleaves to significantly reduce the damage area during impact events, their effect on the residual compressive strength is much less investigated. Among the few published studies on CAI strength of nanofiber interleaved composites, it is notable that a reduced damage area does not always translate to an improvement in CAI strength [1,6,27]. In some cases, a 60% reduction in the projected damage area may not even lead to any enhancement in CAI strength [1,6]. However, one study reported a 19% increase in residual compressive strength when using PA6.6 nanofibers [31]. A distinguishing feature of that study is the use of ±45° biaxial stitched fabrics in the lay-up, which introduces additional tortuosity in the interlaminar regions [36]. These findings highlight the need for a broader study of the relationship between lay-up, damage reduction, and CAI strength in nanofiber interleaved composites.

Therefore, in this study, three relevant lay-up configurations—cross-ply [0°/90°]2s, quasi-isotropic [0°/45°/90°/−45°]s, and all-woven [(0°,90°)]4s—are examined side by side to investigate the role of ply orientation and ply structure on the impact damage resistance and residual compressive strength of nanofiber–interleaved composites. These three lay-up configurations—cross-ply, quasi-isotropic, and all-woven—were selected to represent a broad range of composite architectures and assess how nanofiber toughening interacts with different ply orientations and interlaminar structures. The woven lay-up was chosen for its balanced in-plane properties and inherent tortuosity, while the cross-ply and quasi-isotropic configurations were selected for their distinct fiber orientations and smooth interlaminar regions.

Virgin and nanofiber-toughened composite plates were fabricated using PEBA nanofibrous membranes as interleaves, selected for their high interlaminar fracture toughness, superior performance across various laminate structures, and significantly higher temperature resistance [37,38]. The focus is on carbon fiber–epoxy composites due to their relevance for the automotive and especially aerospace industries, where the compression strength after impact is a very important property in design. Additionally, the brittle nature of carbon fibers makes them particularly susceptible to sudden failure after impact, highlighting the importance of improving their damage tolerance. Specimens were cut from these plates and subjected to flexural testing, interlaminar shear testing, and low-velocity drop-weight impact tests. The flexural and interlaminar shear strength were used to assess the overall quality of the different plates after manufacturing. The projected damage area of the impacted samples, with and without nanofibers, was analyzed using C-scans for each lay-up configuration, and microscopy was employed to further comprehend the findings. Finally, the residual strength of the impacted specimens was evaluated through Compression After Impact (CAI) tests.

## 2. Materials and Methods

### 2.1. Nanofiber Interleave Production

The process for electrospinning PEBA nanofibers is described in detail in ref. [38]. In summary, 8 wt% PEBA (PEBAX72, Arkema, Paris, France) was dissolved in a 60/40 mixture of formic acid and anisole (Sigma-Aldrich, Darmstadt, Germany). PEBAX72 has proven to result in high improvements in interlaminar fracture toughness [37,38]. The datasheet of the bulk polymer lists the following properties: Young’s modulus around 500 MPa, elongation at break up to 300%, an ultimate tensile strength around 55 MPa, and a melting temperature of 180 °C. Homogeneous stand-alone sheets of nanofiber veils, with a surface density of 5 gsm, are fabricated using an in-house developed multi-nozzle electrospinning setup. The setup employs 8 nozzles, with a voltage difference of 30 kV between the nozzle tips and the collector, and a solution flow rate of 2 mL/h per nozzle. The resulting nanofibers have an average diameter of 160 ± 30 nm.

### 2.2. Composites Production

**Figure 1:**
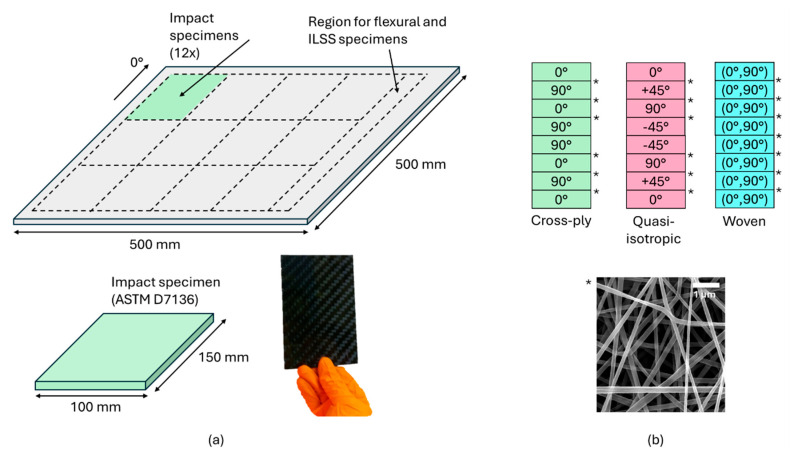
(**a**) Geometry of the manufactured composite plates and the specimen layout. (**b**) Nanofiber veils are interleaved between the reinforcing plies of three configurations (interlayers marked with ‘*’), resulting in toughened composites.

Three different composite lay-ups were fabricated, both with and without nanofiber interleaves (Figure 1). The cross-ply [0°/90°]2s and quasi-isotropic [0°/45°/90°/−45°]s configurations utilized unidirectional carbon fiber fabrics (400 g/m^2^, Zoltek™ PX35 carbon fiber, Zoltek, Bridgeton, NJ, USA), while the woven fabric lay-up [(0°,90°)]4s employed a carbon twill weave fabric (400 g/m^2^, Aksaca A-49 carbon fiber, 12 k 800 tex 2 × 2 twill weave, DowAksa, Akasya, Turkey). Both fabric types were sourced from R&G Faserverbundwerkstoffe GMBH (Waldenbuch, Germany). The nanofiber interleaves, with an areal density of 5 g/m^2^, which has previously been reported to be a well-suited amount to improve delamination and impact resistance [6,21], were carefully positioned on top of the reinforcing plies during the lamination process. In the cross-ply and quasi-isotropic configurations, nanofibers were interleaved at each interlayer except on the midplane (six in total), while in the woven fabric lay-up, nanofibers were interleaved between each of the seven interlayers. Expressed to the total mass of the composite, the mass percentage of nanofiber interleaves added is only around 0.5–1%.

Laminates incorporating nanofibers in the interlayer, as well as non-toughened laminates without nanofiber reinforcements, were fabricated using Vacuum Assisted Resin Infusion. The epoxy preparation and impregnation followed the procedure described in Ref. [37]. The epoxy resin (EPIKOTE MGS RIMR135) and hardener (EPIKURE MGS RIMH137), supplied by Momentive (New York, NY, USA), were used in a 100:30 mass ratio. A curing step was conducted at 80 °C for 5 h immediately after resin impregnation.

The total laminate thickness was measured using a caliper at a minimum of three different locations on each impact sample, resulting in at least 36 measurements for each of the six configurations (three different lay-ups, with and without nanofibers).

### 2.3. Flexural and Interlaminar Shear Strength Testing

Flexural testing of the composites was performed in accordance with ASTM D7264 [39] to evaluate the flexural strength of the produced plates. Specimens were cut into rectangular bars with dimensions of 153 × 13 mm, and tests were conducted using a three-point bending fixture with a span-to-thickness ratio of 32:1, on an electromechanical universal testing machine (Instron 3369, Norwood, MA, USA) equipped with a 2 kN load cell. The load was applied at a constant rate until specimen failure, and the flexural properties were calculated from the load-deflection data.

Interlaminar shear strength (ILSS) testing was conducted following ASTM D2344 [40]. The specimens were prepared with a span-to-thickness ratio of 4:1 and tested using a three-point bending setup. The maximum load before failure was used to calculate the ILSS.

### 2.4. Low-Velocity Impact Testing

Drop-weight impact tests were conducted on the laminates in accordance with the ASTM D7136 [41] standard, which outlines the procedure for evaluating the damage resistance of fiber-reinforced polymer composites subjected to a concentrated, low-velocity impact. The tests were performed over a range of impact energies, specifically targeting the energy levels around the Barely Visible Impact Damage (BVID) threshold. The BVID threshold is defined as the energy level at which a permanent indentation of less than 300 μm is formed on the surface of the laminate, indicating the onset of barely visible damage [21].

**Figure 2:**
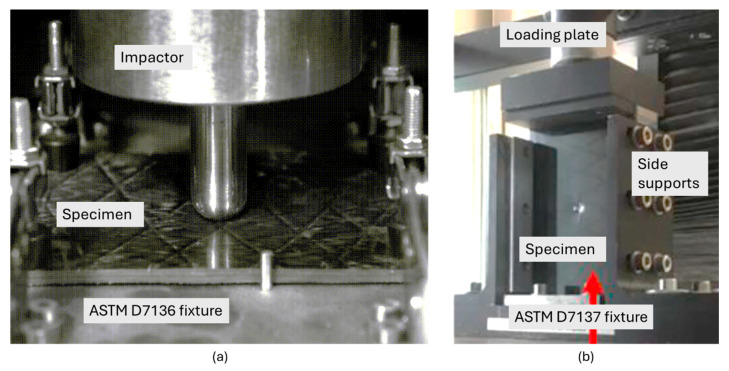
(**a**) Still image taken by the high-speed camera during the low-velocity impact test according to ASTM D7136. (**b**) Compression-after-impact testing according to the ASTM D7137 [42] standard.

A guided impactor, equipped with a dynamically rated load cell, position sensor, and accelerometer, is dropped from a predetermined height to deliver the specified impact energy to the composite specimen. The impactor used in this study had a hemispherical tip, designed to simulate typical out-of-plane impact events, such as those caused by tool drops or debris strikes. The impact energies were carefully selected to span the range around the BVID threshold, ensuring that both sub-threshold (no visible damage) and supra-threshold (visible damage) energies were included. The impact event was also recorded using a high-speed camera (Photron SA4, Tokyo, Japan) to evaluate the test validity (Figure 2a). An anti-rebound device was used to avoid a second strike of the impactor on the specimen.

Specimens of 150 × 100 mm^2^ were cut from the composite plates in accordance with the standard. Three specimens per impact energy level were considered. After impact, the extent of damage was assessed using a depth gauge and ultrasonic C-scanning to quantify the size and severity of the damage. The damage area is measured by ultrasonic C-scanning the full sample of 100 × 150 mm^2^ using a 5 MHz transducer. The scanning resolution was 0.1 mm over the long axis of the sample and 0.5 mm over the short axis of the sample. The projected damage area is calculated from the area where the transmission amplitude drops by 3 dB (50% amplitude reduction).

One specimen for each configuration and at each impact energy level was used for microscopic analysis using optical microscopy (Olympus BX51, Olympus, Tokyo, Japan) and scanning electron microscopy (Phenom XL, Thermo Fisher Scientific, Waltham, MA, USA). The impacted specimens were cut with a diamond saw in the width direction near the impact point. After polishing, the cut edge was inspected using microscopy to assess the different damage mechanisms. A relatively large region of interest was imaged using scanning electron microscopy by an automated image-stitching algorithm, which allowed it to cover a large surface area with a high amount of detail. These stitched images were used to analyze the damage profile in the specimens.

### 2.5. Compression After Impact Testing

Following the impact tests, the laminates were evaluated for their residual compressive strength, commonly referred to as Compression After Impact (CAI) strength, in accordance with the ASTM D7137 standard (Figure 2b). The CAI tests were conducted using an electromechanical Instron universal testing machine. The compressive load was applied at a controlled rate until the specimen failed. Specimens that exhibited edge failure, an invalid failure mode according to ASTM D7137, were excluded from the analysis to ensure the accuracy and reliability of the results. The CAI strength was calculated by dividing the maximum load at failure by the cross-sectional area of the specimen.

## 3. Results and Discussion

### 3.1. Laminate Quality and Low-Velocity Impact Response

**Figure 3:**
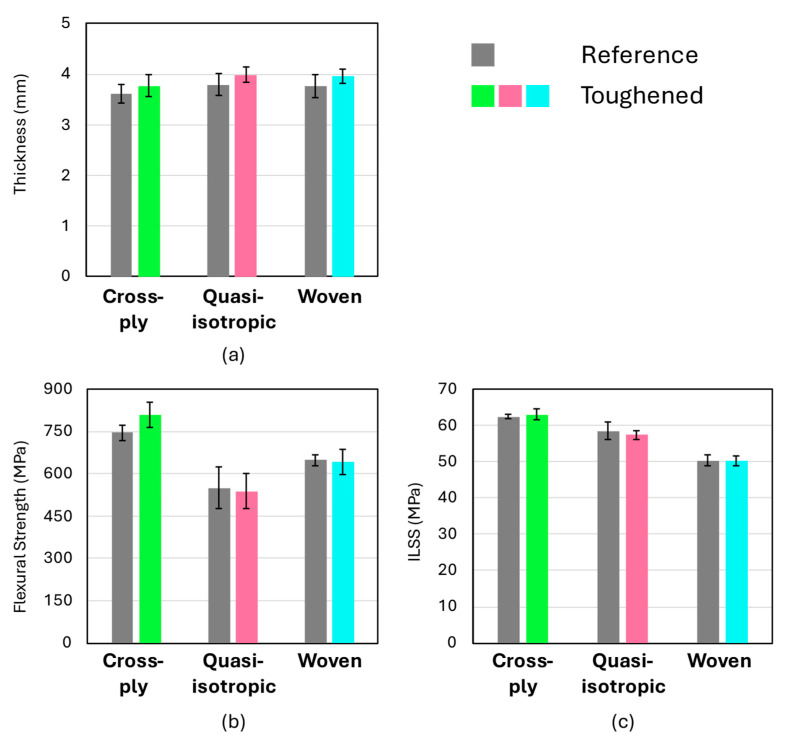
(**a**) Thickness, (**b**) flexural strength, and (**c**) ILSS of the produced composite laminates for both the reference configuration (without nanofibers) and the nanofiber toughened configuration.

Specimens for flexural and interlaminar shear strength (ILSS) testing were cut from the same composite plates used for the impact specimens to evaluate the overall quality of the laminates. The introduction of toughened interlayers can potentially reduce these mechanical properties if the interlaminar regions become excessively thick [43]. However, as shown in Figure 3, the results demonstrate that the toughened composites do not exhibit any significant changes in flexural strength or interlaminar shear strength compared to the non-toughened counterparts.

The toughened laminates were found to be marginally thicker than the non-toughened ones, which is attributed to the presence of the toughened interlaminar regions. Specifically, for all three configurations, the laminate thickness increased by approximately 200 µm on average, with the nominal thickness of the reference configurations being around 3.7 mm (Figure 3). For a nanofiber veil with an areal density of approximately 5 gsm, the expected interlaminar region thickness would be around 30 µm [21]. Thus, the observed increase of 200 µm aligns well with expectations, considering that each configuration contains 6 (cross-ply, quasi-isotropic) or 7 (woven) toughened interlayers.

**Figure 4:**
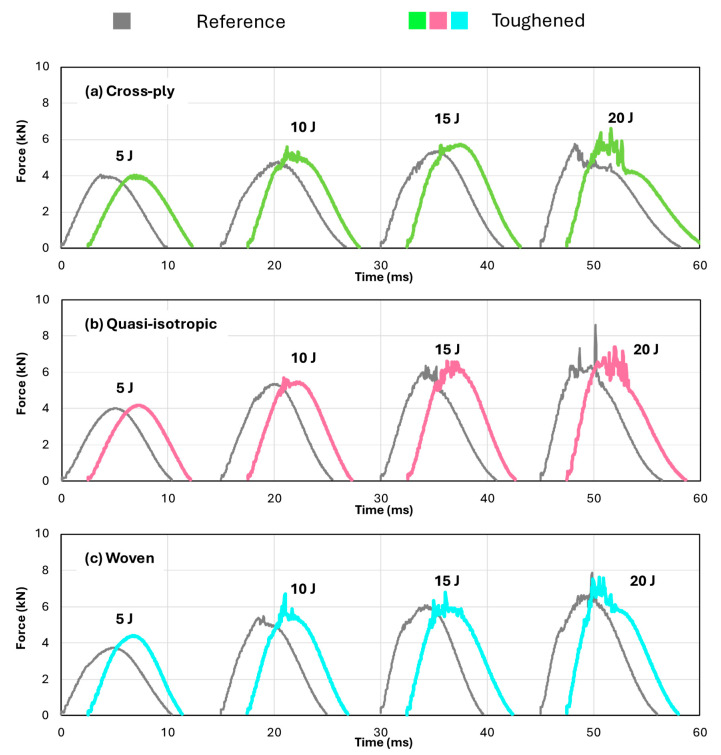
Representative force versus time curves of the impact tests on reference (in grey) and toughened (in color) specimens. The curves are offset on the time axis for illustration purposes only.

Figure 4 presents representative curves of the reaction force measured on the impactor as a function of time for both the reference (depicted in grey) and nanofiber-toughened (depicted in color) specimens, impacted at four different energy levels. For clarity, the curves are offset along the time axis. The overall shape of the curves aligns with expectations, showing higher reaction forces and increased damage, indicated by fluctuations in the force signal near the peak force, at higher impact energies. The differences observed between the reference and toughened specimens at the same impact energy are minimal. However, the toughened specimens tend to exhibit a slightly higher peak force and a shorter duration of the impact event, suggesting a potential improvement in damage resistance.

### 3.2. Damage Area in Toughened and Non-Toughened Specimens After Impact

**Figure 5:**
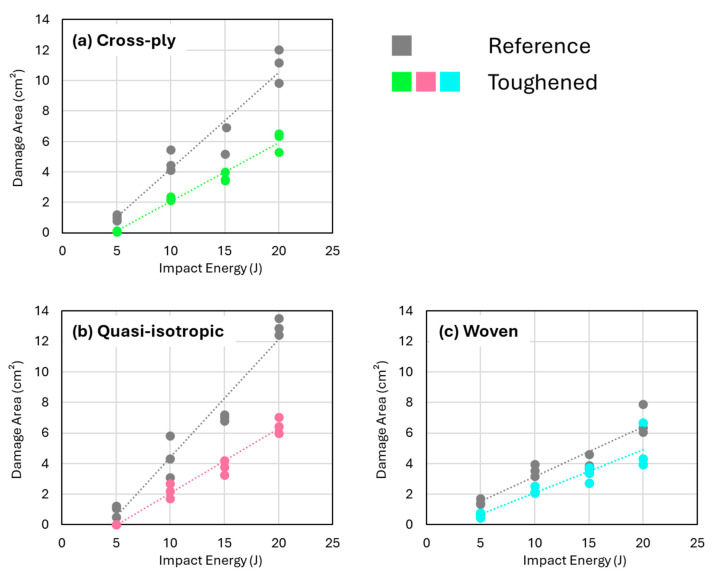
Projected damage area versus impact energy for (**a**) cross-ply, (**b**) quasi-isotropic, and (**c**) woven composites show a clear reduction in damage area upon interleaving the composites with nanofibrous veils. A linear fit is plotted for illustration purposes.

Figure 5 shows the projected damage area (obtained from C-scanning) plotted against the impact energy level for each of the three configurations. The integration of nanofibers within the composites demonstrated a significant reduction in projected damage area for each lay-up configuration across the considered impact energy range. It should be noted that the linear trendlines presented in Figure 5 are provided for indicative purposes and do not necessarily imply a true linear relationship between impact energy and the projected damage area. From these curves, it is clear that the presence of the nanofibrous veils results in an improved impact damage response irrespective of the specific lay-up.

The graphs of projected damage area versus impact energy show nanofiber-toughened laminates with a quasi-isotropic and cross-ply layup have a noticeably lower slope for the trendline than those of the non-toughened references, indicating a more effective energy absorption and damage mitigation. The woven fabric laminates show a much smaller reduction in projected damage area for the toughened composites, but they also have the smallest overall projected damage areas in both toughened and non-toughened states. This aligns with the literature, where it is also stated that fabrics are preferred over unidirectional lay-ups for improved impact resistance [10]. These results suggest that the composite architecture itself may play a role in how effective the nanofibers are in reducing damage. This is in line with our previous results on nanofiber toughening in composites with dissimilar interfaces tested using Mode I and Mode II delamination tests [37], where the toughening efficiency was also heavily influenced by ply architecture.

**Figure 6:**
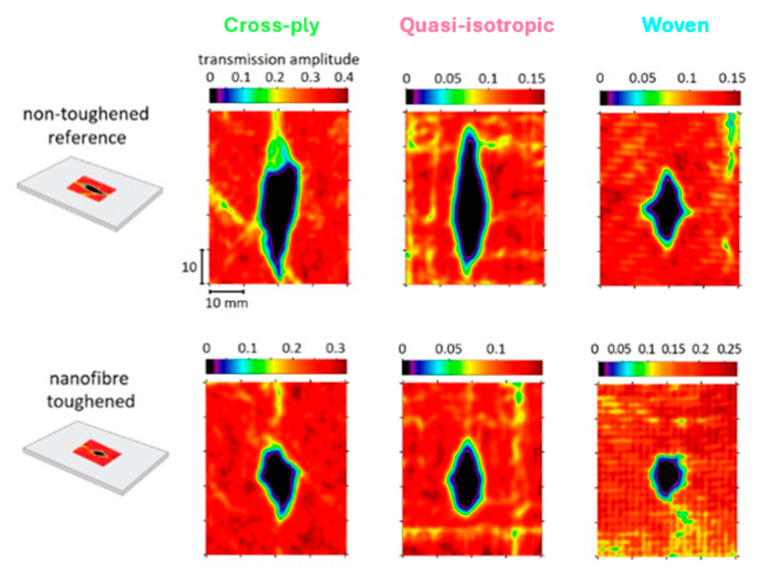
C-scan images of the different specimens impacted at 20–25 J showing a rhombus-like projected damage area with a clear reduction in projected damage area for the toughened specimens.

**Figure 7:**
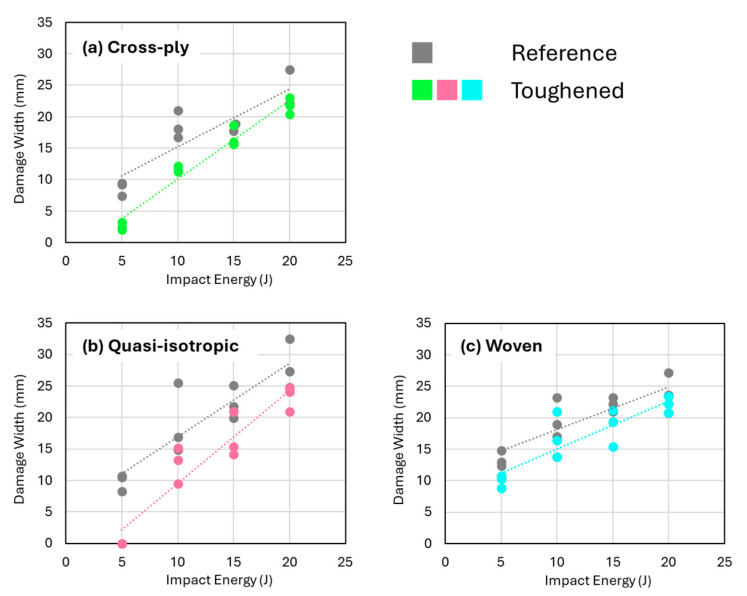
Damage width versus impact energy for the (**a**) cross-ply, (**b**) quasi-isotropic, and (**c**) woven configurations. The difference between toughened and reference specimens is less extensive in comparison to the projected damage area.

The C-scan images reveal that the projected damage area is more localized when nanofibers are present (Figure 6). Interestingly, this reduction in projected damage area seems to be mainly due to a localization in the longitudinal direction (longest sample direction). The damage width seems to be less influenced by the presence of nanofiber interleaves, especially at the highest impact energies (Figure 7). This suggests that the sample geometry, prescribed by the ASTM D7136 standard, can also influence the specific toughening mechanisms acting during the low-velocity impact event, as longitudinal damage growth seems to be preferred in this setup.

**Figure 8:**
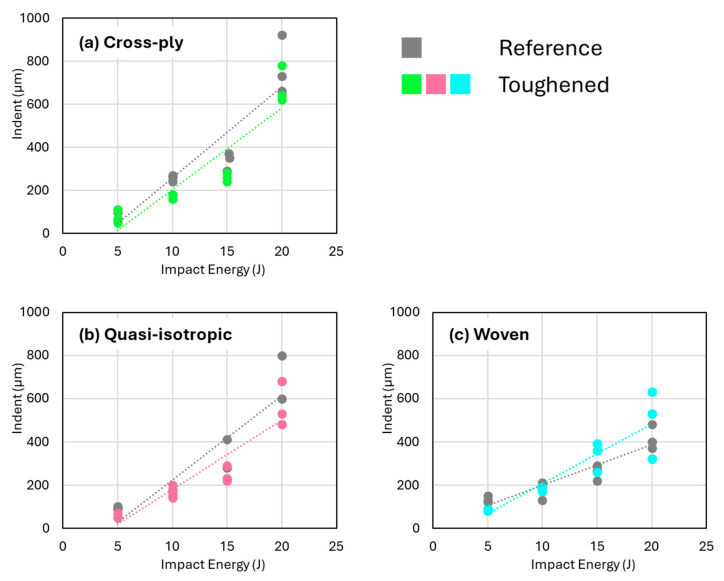
Indent depth versus the impact energy for (**a**) cross-ply, (**b**) quasi-isotropic, and (**c**) woven impacted specimens. No significant differences are observed between reference and toughened specimens.

While nanofiber toughening significantly reduces the projected damage area across all three studied laminate architectures, it is noteworthy that the depth of the resulting indent remains relatively consistent between nanofiber-toughened and non-toughened samples at comparable impact energies (Figure 8). Despite the toughened specimens exhibiting more localized damage, as evidenced by the C-scan images, this concentration of damage did not lead to a deeper dent in the specimens. This suggests that while nanofibers effectively confine the impact damage, the overall degree of fiber failure and epoxy plasticity/fracture at the impact site—and thus the “barely visible impact damage” (BVID) characteristic—remain largely unchanged.

### 3.3. Cross-Sectional Examination of Impacted Specimens

**Figure 9:**
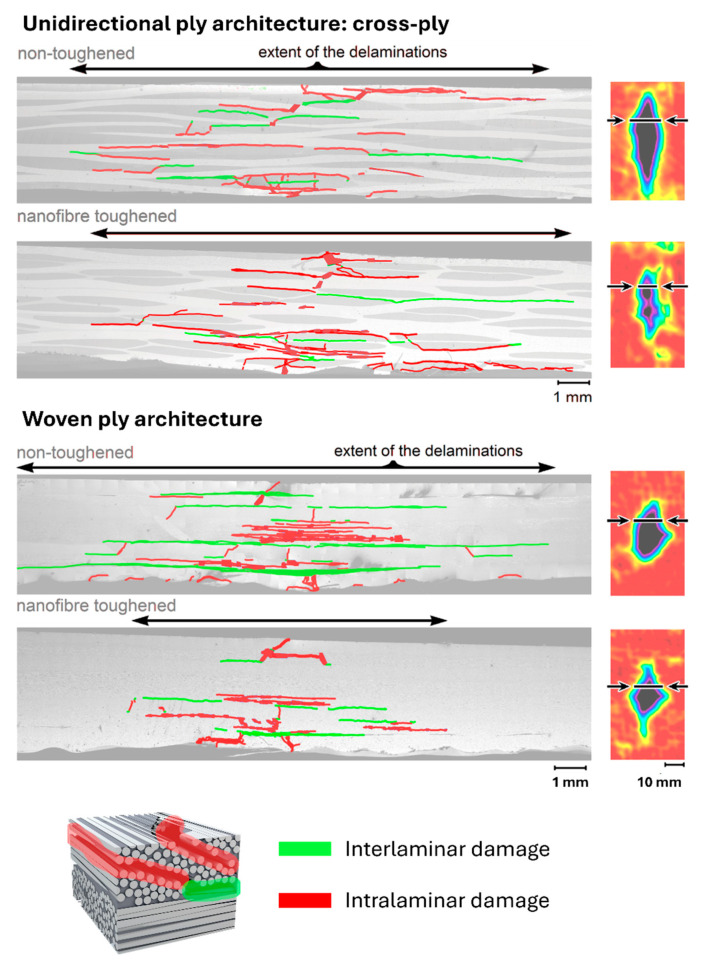
Stitched SEM images of cross-ply and woven fabric composites impacted at an energy of 15 J. Interlaminar cracks and delaminations are marked in green, while intralaminar cracks and damage are marked in red.

**Figure 10:**
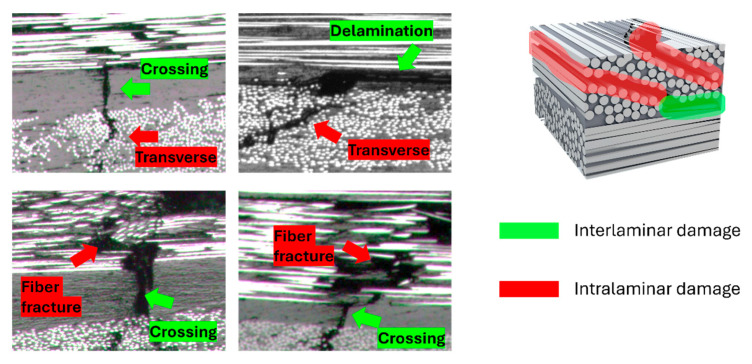
Overview of observed interlaminar and intralaminar damage mechanisms used to create the damage profiles in Figure 9.

Figure 9 presents SEM images of the specimens’ cross-sections taken near the edge of the impact damage area, showcasing two different composite architectures: a lay-up composed of unidirectional plies represented by the cross-ply stacking and an all-woven fabric lay-up. The cross-sectional images were analyzed at high resolution, with various types of microscale damage, including fiber fractures, delaminations, and other cracks. The damages were then color-coded by their spatial occurrence as either interlaminar damage (delaminations, interlaminar cracks) or intralaminar damage (fiber fracture, transverse cracks, debonding) to visually assess the overall damage profile in the different specimens. Figure 10 gives an overview of the different damage mechanisms found in the interlaminar and intralaminar regions of the composites.

In the cross-ply specimens, the presence of nanofibers has led to a notable reduction in interlaminar damage (highlighted in green). The nanofibers effectively hinder crack propagation between the relatively smooth interlayers, fulfilling their intended role. However, these cross-sections also indicate that the extent of intralaminar damage (highlighted in red) remains largely unaffected by the introduction of nanofibers. Thus, while nanofibers significantly reduce interlaminar delaminations, they do not appear to alter the other damage phenomena. In contrast, for the woven configurations, both a decrease in interlaminar damage (highlighted in green) as well as intralaminar damage (highlighted in red), can be observed in the toughened specimens.

### 3.4. Compression After Impact Strength

**Figure 11:**
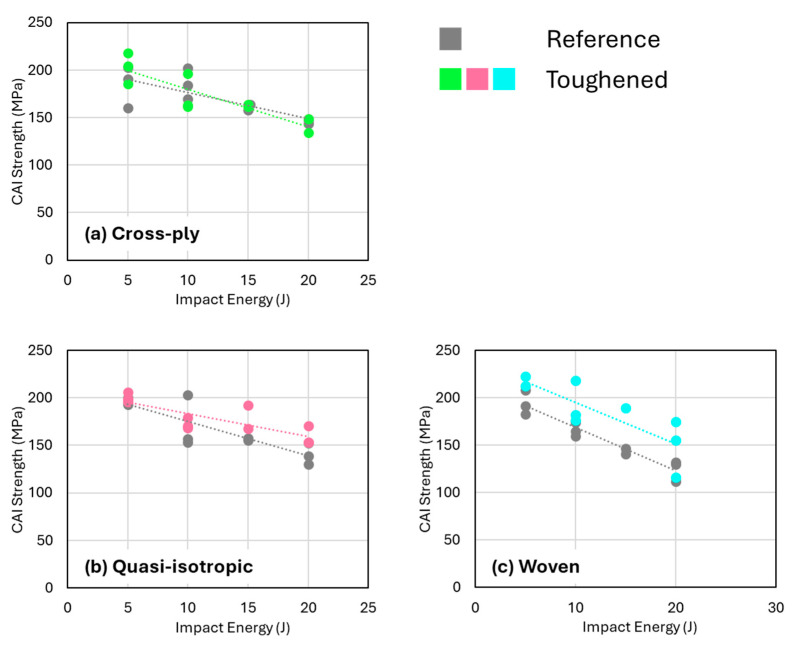
CAI strength versus impact energy for the (**a**) cross-ply, (**b**) quasi-isotropic, and (**c**) woven configurations. Only for the woven specimens a significant increase in CAI strength is observed.

Figure 5 showed that quasi-isotropic and cross-ply samples exhibited the largest reductions in projected damage area as detected by C-scans, especially when compared to the woven fabric composites. However, this trend is completely different for the residual compressive CAI strength, as shown in Figure 11. Surprisingly, the quasi-isotropic and cross-ply composites do not demonstrate any improvement in CAI strength, despite the significant reductions in projected damage area attributed to the presence of nanofibers. In contrast, the woven fabric composites show a notable increase in CAI strength when interleaved with nanofibers. According to the trendlines in Figure 11, the CAI strength of woven fabric composites increases by approximately 13% at 5 J compared to non-interleaved reference samples, and by around 26% at 22 J.

The differing impact behavior between woven fabric-reinforced composites toughened with nanofibers and multidirectional architectures composed solely of unidirectional plies can be attributed to the distinct nature of the damage profile in these composites. In woven fabric composites, as discussed in relation to Figure 9, nanofiber toughening effectively reduces both interlaminar and intralaminar damage. In contrast, in multidirectional lay-ups, the nanofibers primarily reduce interlaminar delaminations while leaving the degree of intralaminar damage largely unchanged. A possible explanation for this is that in the samples with unidirectional plies, the toughening effect of the nanofibers leads to localized absorption of the impact energy, initiating other damage mechanisms, such as fiber fracture and transverse cracking in the intralaminar regions, ultimately leading to a CAI lower than expected. For the woven specimens, the projected damage area decreases to a lesser extent than for the other configurations; however, both interlaminar delaminations and intralaminar damage are reduced more uniformly.

**Figure 12:**
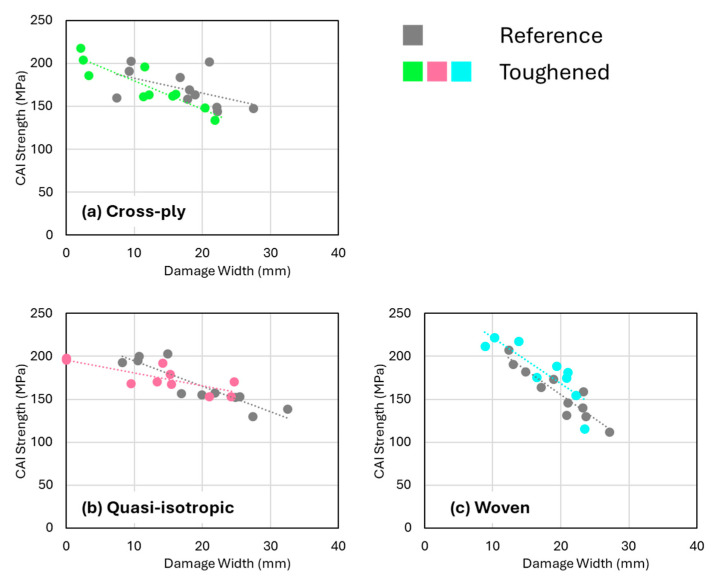
CAI strength versus damage width for the (**a**) cross-ply, (**b**) quasi-isotropic, and (**c**) woven specimens.

The somewhat unexpected observation that nanofibers significantly reduce impact-induced delaminations without improving CAI strength suggests that the delaminations (present in the central region of the specimens) may not critically affect the overall compression strength in comparison to the intralaminar damage. Research indicates that the impact of delaminations on compression strength is complex and depends—among other variables—on the geometry, ply orientation, and type of delaminations [44,45,46,47,48,49,50]. Particularly, embedded delaminations, which most closely resemble those caused by low-velocity impacts in this study, may have little to no effect on compression strength if they remain below certain size thresholds [51], typically around 20% of the plate’s total length and width. This is about 20–30 mm for the ASTM D7136 specimen used in this study, indeed corresponding to be within the range of the observed damage widths. This suggests that in both nanofiber-toughened and non-toughened composites, the delaminations observed after low-velocity impacts are likely not the primary factor limiting CAI strength. In addition, when the CAI strength is plotted versus the observed damage width from the C-scan data, the data points tend to fall on the same trendline irrespective of the presence of toughened interlayers (Figure 12). This indicates that the degree of intralaminar damage, or at least the combination of both interlaminar and intralaminar damage, is important for the CAI strength and that a reduction in only the delamination area does not necessarily improve the CAI strength.

## 4. Conclusions

This study demonstrates the effectiveness of PEBA nanofibers in significantly reducing the impact damage area in three different composite lay-up configurations: quasi-isotropic, cross-ply, and stacked woven fabrics. The flexural strength and interlaminar shear strength of the laminates remained unaffected by the addition of nanofibrous interleaves: around 750 MPa and 63 MPa for cross-ply, 550 MPa and 58 MPa for quasi-isotropic, and 650 MPa and 50 MPa for woven laminates, respectively. After low-velocity impact testing in a range of 5–20 J, the observed reduction in projected damage area, ranging from 30% to 50%, aligns well with findings from previous studies involving other polymer nanofiber interleaves. An improved delamination resistance is obtained due to the presence of tough nanofibers in the interlayers, resulting in a smaller damage area upon impact loading. Despite the nanofibers’ capacity to absorb energy during delamination, their influence on subsequent CAI strength appears to be limited. For the cross-ply and quasi-isotropic specimens, the CAI strength decreased from about 200 MPa at 5 J impact to 150 MPa at 20 J impact, and there is no significant toughening effect of the nanofibrous interleaves. Only the woven fabric composites exhibited a positive effect of nanofiber toughening on CAI strength over the full impact energy range of approximately 12%. The reference configuration had a CAI strength that decreased from 200 MPa at 5 J to about 125 MPa at 20 J, while the nanofiber toughened specimens showed a CAI strength of 220 MPa at 5 J to around 150 MPa at 20 J. This improvement is attributed to the observation that a reduction in interlaminar as well as intralaminar damage is obtained for the toughened specimens.

Cross-sectional analysis of the impacted composites revealed that while multidirectional lay-ups with unidirectional plies also experienced a substantial reduction in delamination area, this did not correspond to a reduction in intralaminar damage. This finding suggests that intralaminar damage, rather than delamination size, plays a key role in the composites’ susceptibility to failure under compressive loads. Indeed, buckling theory indicates that, in the low-velocity impact scenarios considered, delaminations can be insufficient in size to induce buckling of the sub-layers. As a result, delaminations are not the dominant factor in initiating damage during compression. Hence, the improved delamination resistance in the nanofiber-toughened specimens does not necessarily lead to a better residual compressive strength after impact.

## Data Availability

The original contributions presented in the study are included in the article, further inquiries can be directed to the corresponding author.
